# HSF1: Primary Factor in Molecular Chaperone Expression and a Major Contributor to Cancer Morbidity

**DOI:** 10.3390/cells9041046

**Published:** 2020-04-22

**Authors:** Thomas L. Prince, Benjamin J. Lang, Martin E. Guerrero-Gimenez, Juan Manuel Fernandez-Muñoz, Andrew Ackerman, Stuart K. Calderwood

**Affiliations:** 1 Department of Molecular Functional Genomics, Geisinger Clinic, Danville, PA 17821, USA; 2 Department of Radiation Oncology, Beth Israel Deaconess Medical Center, Harvard Medical School, Boston, MA 02115, USA; 3 Laboratory of Oncology, Institute of Medicine and Experimental Biology of Cuyo (IMBECU), National Scientific and Technical Research Council (CONICET), Buenos Aires B1657, Argentina

**Keywords:** heat shock factor 1, HSF1, cancer, heat shock proteins, HSPs, molecular chaperones, heat shock response, HSR, HSF1 in cancer, metastasis, cancer therapy, tumorigenesis, HSF1 expression in cancer

## Abstract

Heat shock factor 1 (HSF1) is the primary component for initiation of the powerful heat shock response (HSR) in eukaryotes. The HSR is an evolutionarily conserved mechanism for responding to proteotoxic stress and involves the rapid expression of heat shock protein (HSP) molecular chaperones that promote cell viability by facilitating proteostasis. HSF1 activity is amplified in many tumor contexts in a manner that resembles a chronic state of stress, characterized by high levels of *HSP* gene expression as well as HSF1-mediated non-*HSP* gene regulation. HSF1 and its gene targets are essential for tumorigenesis across several experimental tumor models, and facilitate metastatic and resistant properties within cancer cells. Recent studies have suggested the significant potential of HSF1 as a therapeutic target and have motivated research efforts to understand the mechanisms of HSF1 regulation and develop methods for pharmacological intervention. We review what is currently known regarding the contribution of HSF1 activity to cancer pathology, its regulation and expression across human cancers, and strategies to target HSF1 for cancer therapy.

## 1. Introduction

The heat shock response is one of the most explosive biochemical events experienced in mammalian cells [1]. The ability to adapt to changes in environmental thermal energy is fundamental to the survival of all cellular organisms. When cells are exposed to stresses, such as elevated temperatures, that compromise the structural integrity of the proteome, a cohort of HSP molecular chaperone proteins are rapidly and abundantly expressed [1,2,3]. Such molecular chaperones function by physically interacting with other proteins to fold, maintain, and restore their three-dimensional structure or direct them for degradation [4,5]. In eukaryotes the expression of *HSP* genes is coordinated by HSF1. The guardian of the proteome, HSF1, once activated, swiftly binds to heat shock elements (HSEs) in the promoters of *HSP* genes, initiating synchronous transcription of these previously silent genes [6,7]. In this way, HSF1 allows adaptation to acute environmental stress by increasing the protein-folding capacity of the cell, a response that also endows the cell with greater resilience to subsequent stress. However, activation of HSF1 also promotes HSP expression in cancer cells that together with other HSF1 activities facilitate tumor cell survival, resistance, and enables malignant cell growth.

Apparent constitutive activation of the HSR pathway in cancer was first indicated by studies in the 1980s that found altered levels of HSPs in transformed cells, as reviewed in [8,9]. The extent to which HSP expression is altered in specific cancer types has since been shown to apply differentially across specific *HSP* family members and was recently shown at the transcriptomic level in human breast tumors [10]. HSPs play a multitude of roles in tumorigenesis, properties that have underscored the importance of understanding the basis of HSR activation in human cancers, as reviewed in [11,12]. 

The importance of the HSR is underscored by the existence of more than five HSF paralogs in humans [13], although only *HSF1, HSF2*, and *HSF4* have been reported in the cancer literature. HSF2 influences neurodevelopment and is able to amplify the HSR in collaboration with HSF1 [14,15,16]. Inhibitors that target the proteasome or the N-terminus of Hsp90 induce the expression of HSF2 [17,18]. Increased expression of *HSF2* is observed in lung cancer samples along with increased HSP levels [19]. Conversely, in prostate cancer, HSF2 suppresses tumor invasion by promoting acinar morphogenesis [20]. *HSF4* encodes two isoforms with Hsf4a generally considered to restrict HSF1 transactivational activity [21,22], while the major isoform Hsf4b promotes Hsp70 expression in a cell cycle-dependent manner [23].

Despite the presence of several paralogs, HSF1 is the primary factor that executes activation of the HSR. This primacy was first demonstrated in a study by McMillan et al., showing that HSF1 deletion abolishes HSR responsiveness to acute stress due to a lack of induction of HSP expression and this deficit was coupled with increased sensitivity to stress insults [24]. HSF1 is therefore generally considered the most robust regulator of HSP expression and the major mediator of increased HSPs in cancer. The majority of the available information related to HSP expression from both the stress response and cancer fields involves HSF1, and we have therefore concentrated on this principal transcription factor [13]. 

**Figure 1:**
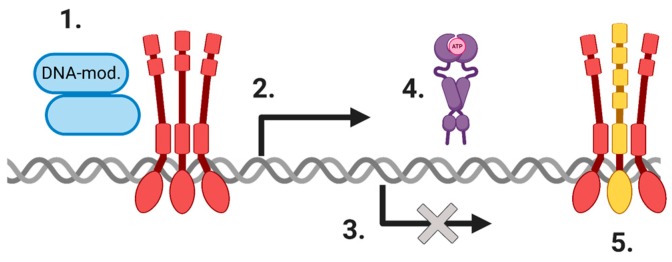
The general mechanisms by which HSF1 affects its biological activities. Shown here to include (**1**) Recruitment of DNA-modifying proteins, such as HDACs, the SWI/SNF complex, DNMT3a, or MTA1, (**2**) Transcriptional activation of gene targets, (**3**) Repression of transcription, (**4**) Processes regulated by HSF1 gene targets, such as Hsp90, (**5**) Co-operation with other transcription factors, including HSF2 depicted in yellow. Figure created with Biorender.com.

Activation by proteotoxic stresses, such as heat shock, induces HSF1 to transition from a monomer to trimer, translocate into the nucleus, locate *HSP* gene promoters, and activate transcription within 30 s, a time period that we consider to be as quick as heat shock can be experimentally delivered and measured [25,26,27]. This process has endowed eukaryotes with the ability to evolve within changing environments. Trimerization of HSF1 is mediated by hydrophobic repeat regions (HR-A, HR-B, and HR-C), also described as leucine zipper domains (LZ-1, LZ-2, LZ-3, LZ-4), that mediate a parallel arrangement of HSF1 monomers into activated trimers. This mechanism allows each DNA-binding domain (DBD) located at the N-terminus to be orientated next to the other (Figure 1) [6]. Each monomer of the HSF1 trimer binds to a 5′-nGAAn-3′ unit within a heat shock element (HSE) motif found near the transcription start site of *HSP* genes [28]. The winged helix-turn-helix DBD is the most well-conserved and structured domain across all HSF orthologs [6,29,30,31]. This conservation contrasts with the remainder of the HSF1 sequence, which is predominantly unstructured but interspersed with some regions of higher order [31,32], a characteristic of many key regulatory factors that interact with numerous other proteins [33]. Consequently, attempts to crystallize the full-length tertiary structure of human HSF1 have not succeeded to our knowledge, making it difficult to determine precisely the relationships between the HSF1 structure and biological function for these latter regions. Upon the binding of an active HSF1 trimer to an HSE close to the transcriptional start site, transcription is activated by twin carboxyl-positioned transactivating domains (TADs), which are required for HSF1-mediated transcriptional activation [34]. A centrally positioned regulatory domain (RD), which is capable of sensing heat and activating transcription [35], is subject to extensive post-translational modifications and is believed to regulate an intramolecular fold of inactive HSF1 monomers between the HR-C and HR-A/B. The formation of an active HSF1 trimer, its binding to DNA, and transcriptional activation appear to be regulated by a combinatorial series of post-translational modifications [36]. It seems likely that the mechanisms of HSF1 activation, including the activity of upstream regulatory molecules, will differ qualitatively and quantitatively in transformed cells in which, instead of the acute and transient induction observed after stress, HSF1 becomes switched on chronically in a mechanism unlikely to involve acute proteotoxic stress [1,26,37,38].

The mechanisms through which HSF1 affects biological activities in the HSR are therefore proposed to be primarily through transcriptional regulation and recruitment of transcriptional and DNA modifying proteins, and extends to the downstream activities performed by the protein products of its target genes (Figure 1). The extent of HSF1 DNA-binding sites and genes subject to HSF1 regulation were shown by several genomic, microarray, and/or transcriptomic studies [39,40,41], the targets of which have recently been collated [42]. 

An important advance gained from these studies was the revelation of the extent of HSF1 transcriptomic negative regulation, as well as a cohort of HSF1 targets in cancer cells distinct to that of heat shock [39,43]. The precise roles of HSF1 in the multiple pathophysiological changes underlying carcinogenesis and tumor progression are still emerging, although numerous studies indicate that HSF1 knockout is effective in inhibiting cancer formation in a range of tissues as shown by several experimental mouse tumor models [44,45,46,47]. Similarly, using data provided by the Cancer Dependency Map (DepMap) study, Dong et al. illustrated the relative high importance of HSF1 to transformed cells compared to other major oncogenic factors [48,49]. Together, these studies demonstrated that cancer cells are frequently dependent upon HSF1 to maintain the transformed state and to form tumors. Several studies have also provided evidence to show that not only is HSF1 essential for transformation but is also hyperactivated and actively promotes enhanced metastatic and/or resistant properties within cancer cells [37,50,51,52]. It should be kept in mind throughout the discussion below that the general mechanisms underlying carcinogenesis are still strongly debated and multiple contrasting theories have been advanced [53,54]. Mechanisms suggested include: (i) A role for sequential gene mutations in initially normal cells leading to stepwise override of cell regulation, (ii) aberrant stem and progenitor cell activity that gives rise to heterogeneous tumor cell populations, (iii) and a pro-tumorigenic interplay between the cellular and molecular components of the tumor microenvironment and cancer cells to drive metastatic and resistant properties in cancer cells and suppress tumor immunity [55,56]. We aim to summarize below what is known regarding the roles of HSF1 in such processes.

## 2. Roles for HSF1 in Cellular Processes Associated with Transformation and the Malignant Properties of Cancer Cells

**Figure 2:**
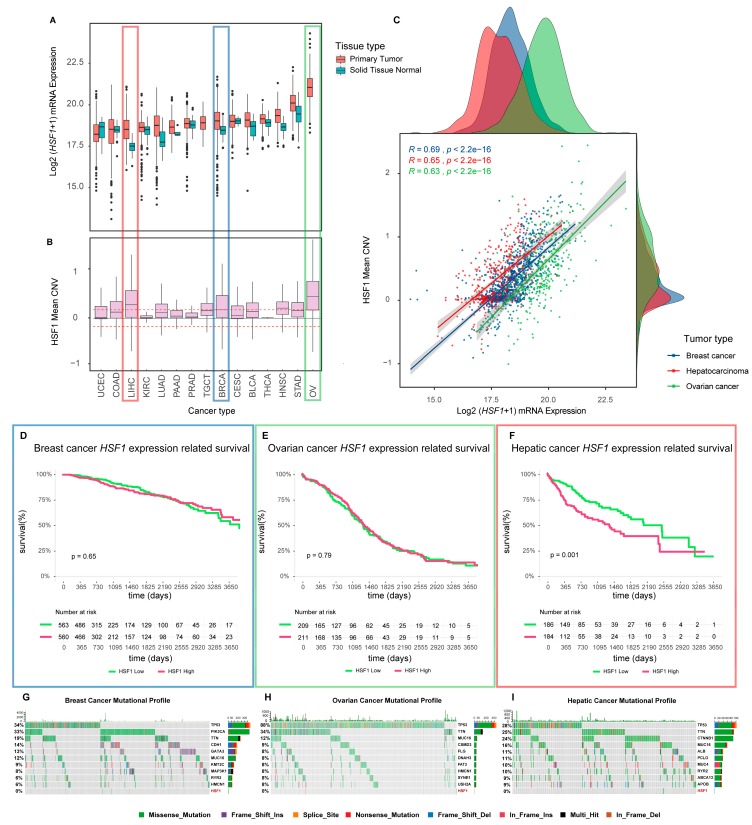
HSF1 mutation, CNV, and mRNA expression profiles across human cancers. (**A**) Box plots of *HSF1* expression across different non-cancerous (blue) and cancerous tissues (red) in the TCGA cohort of cancer (https://gdc.cancer.gov/). RNA-seq mRNA expression values were log2 transformed and depicted to compare its expression distribution in normal and cancer tissues. For testicular germ cell tumors (TGCTs) and ovarian cancer (OV), normal tissue expression was not available. (**B**) Microarray CNV distribution of TCGA cancer samples is depicted in the same order as the expression data to evaluate the CNV occurrence in the different cancer types. Red, blue, and green rectangles indicate the expression and CNV status of liver hepatocarcinoma, breast cancer, and ovarian cancer, respectively. (**C**) Scatter plot and regression lines showing the correlation between CNV and RNA expression in tumor tissues of the three outlined cancer types. On the X and Y outer scatterplot margins, the *HSF1* expression values of each cancer type and distribution of the *HSF1* CNV is depicted, respectively, to assess the relationship between the evaluated values for each cohort. (**D**–**F**) shows Kaplan–Meier plots of the relationship between high and low expression levels of HSF1 in the liver, breast, and ovarian cancer cohorts. The population was divided according to the 50% percentile of HSF1 expression as the cutoff. A log-rank test was used to evaluate the statistical significance of the difference, and survival tables are shown below the plots to evaluate subjects at risk at each time point. (**G**–**I**) are Oncoplots, where each column represents a sample of the corresponding cancer type and each color represents a type of variant. For each cancer, the 10 genes with the highest number of mutations followed by the *HSF1* gene (in maroon) are shown. Above, a small barplot shows the frequency of mutations for each sample, and in the right part, the percentage of samples that have the corresponding mutated gene. Variants annotated as Multi_Hit are those genes that are mutated more than once in the same sample. Abbreviations: CNV: Copy number variation. BLCA: Bladder Urothelial Carcinoma. BRCA: Breast invasive carcinoma. CESC: Cervical squamous cell carcinoma and endocervical adenocarcinoma. COAD: Colon adenocarcinoma. HNSC: Head and Neck squamous cell carcinoma. KIRC: Kidney renal clear cell carcinoma. LIHC: Liver hepatocellular carcinoma. LUAD: Lung adenocarcinoma. OV: Ovarian serous cystadenocarcinoma. PAAD: Pancreatic adenocarcinoma. PRAD: Prostate adenocarcinoma. STAD: Stomach adenocarcinoma. TGCT: Testicular Germ Cell Tumors. THCA: Thyroid carcinoma. UCEC: Uterine Corpus Endometrial Carcinoma.

**Figure 3:**
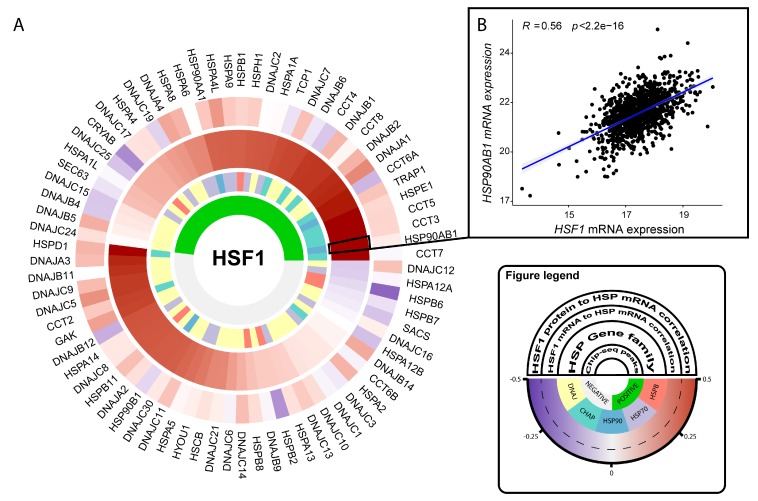
Relationship between *HSF1* mRNA expression, HSF1 protein levels, and promoter occupancy with *HSP* mRNA levels in human breast cancer. (**A**) The circular plot shows the relationship between HSF1 mRNA expression, protein levels, and promoter occupancy across the different *HSP* genes in human breast cancer. In the inner circle, the green blocks show positive ChIP-seq peaks that were detected near the *HSP* genes’ respective regulatory regions (within 2500 bp of the transcription start site (TSS)). The transcription factor binding data was obtained from the UCSC genome browser (http://genome.ucsc.edu/) [69], and is derived from a large collection of ChIP-seq experiments performed by the ENCODE project between February 2011 and November 2018 [70,71]. In the following circle, a color key reflects the different *HSP* gene families according to Kampinga et al. [72]. The last two outer circles depict the Pearson’s correlation coefficients of the relationship between HSF1 (protein and mRNA) expression, and the respective *HSPs* mRNA expression levels in patients’ tumor samples. The patient protein expression data was obtained from the NIH-NCI Clinical Proteomic Tumor Analysis Consortium (CPTAC) data portal (https://cptac-data-portal.georgetown.edu/), specifically from the Cancer Genome Atlas Proteomic dataset. The mRNA expression levels were obtained from The Cancer Genome Atlas [73], downloaded from the NIH Genomic Data Commons portal (https://portal.gdc.cancer.gov/). (**B**) Example scatterplot of the relationship between *HSF1* mRNA expression and *HSP90AB1* mRNA expression.

The first indication that HSF1 might be constitutively active in human tumors was that its gene targets, HSPs, such as Hsp27 and Hsp70, almost exclusively silent in normal tissues, were avidly expressed in breast cancer and other malignancies [8,57,58]. As the most immediate and direct activator of HSP synthesis, it was then hypothesized that HSF1 might be switched on during the development of cancer, and in fact, this was subsequently observed in studies in tissue culture [59,60,61]. More recently, it has been shown that HSF1 is abundant in the nuclei of mammary tumor cells in clinical biopsy samples, indicating constitutive activation of the heat shock response in cancer [38]. Perhaps most significantly, it has been shown that knockout of HSF1 in mouse tumor models, including p53-deficient or Her2-overexpressing spontaneous cancer, severely curtails tumor growth [13,45,46,47,62,63,64]. These studies provide strong evidence for an important causal and essential role for the factor in tumorigenesis. In addition, higher levels of *HSF1* mRNA found in tumor biopsies from a wide range of disease types may indicate a role for the expression of the factor in tumor morbidity (Figure 2A,B) [47,65]. However, only in the case of hepatic cancer did we find a significant correlation between elevated HSF1 gene expression and the survival of patients (Figure 2F) compared to breast and ovarian cancer, in which we could detect no significant relationship (Figure 2D,E). Given that other markers of HSF1 activity, such as CaSig or nuclear localization, are related to breast cancer outcomes [39,52], these findings suggest that the prognostic value of *HSF1* mRNA levels may be specific to different cancer types. While higher levels of *HSF1* expression do positively correlate with HSF1 gene targets in human breast tumor samples (Figure 3), and *HSF1* overexpression in cultured cancer cells can lead to increased HSP levels [61,66], HSF1 activation appears to be primarily regulated at the protein level as may be expected when an immediate transcriptional response is required, such as when the HSR is deployed [67]. Of note, many *HSP* gene levels positively correlate with *HSF1* expression even in the absence of significant levels of HSF1 occupancy within respective *HSP* promoter regions (Figure 3), suggesting the possibility of common upstream regulators, or for *HSF1* expression to be subject to similar epigenetic organizations. Indeed, a common regulator of *HSF1* and a series of key *HSP* genes was recently described for T cell acute lymphoblastic leukemia (T-ALL) in the intercellular-signaling receptor NOTCH1 [47]. Of note, HSF1 protein levels exhibited variable correlative relationships with the mRNA levels of its *HSP* gene targets (Figure 3), possibly further emphasizing the importance of post-translational modifications for HSF1 transcriptional activity, and/or distinct patterns of mRNA metabolism between *HSP* genes. Compared to the most frequently mutated genes across these cancer types, the incidence of HSF1 missense mutations, frame shifts, splice site abnormalities, or frame deletions were minimal (Figure 2G–I). Thus, although *HSF1* mRNA levels were almost universally elevated across cancer types in the TCGA datasets, genetic mutation was rare in the tumors studied. 

The genetic studies performed so far indicate that experimental tumors either do not grow or grow poorly when the *HSF1* gene is inactivated. Tumor growth is the aggregate product of the opposing processes of proliferation on the one hand and death by programmed mechanisms, through necrosis and through senescence, on the other. However, HSF1 does not appear to directly modulate cell growth as *Hsf1*^−/−^ MEFs grew equally well as wild-type cells in vitro [45]. However, the original *Hsf1* KO studies from the Benjamin lab had shown that the factor is required for robust growth of a variety of mouse tissues in vivo [68]. Here, we discuss the roles of HSF1 in influencing tumor growth via modulation of cells’ autonomous changes associated with transformation, such as cell growth and mitosis, cell death, senescence, aneuploidy, metabolism, and differentiation, as well as tissue-level changes in angiogenesis and altered expression of stromal factors, and how these relate to metastasis and resistance. 

### 2.1. HSF1 Promotes Evasion of Cancer Cell Death and Senescence

A key property of cancer cells that enables tumor growth is escape from programmed cell death and senescence that normal cells are subject to. HSF1 is important in the evasion by cells of programmed cell death (PCD) by increasing the levels of HSP27, HSP70, and HSP90, which also function to repress many PCD signals [60,68,74,75,76]. HSF1 may also be required for PCD resistance through its role in expression of the Bag-3 co-chaperone [75], and/or negative regulation of the pro-apoptotic XAF1 [77]. Reduced TUNEL staining and reduced tumor-free survival was reported in *Hsf1+/+ Neu+* experimental tumors compared to *Hsf1+/− Neu*+, indicating higher rates of apoptosis in *Hsf1+/−* tumors [63]. A strong relationship between HSF1 and cell survival was also demonstrated recently in a study by Gaglia et al., whereby the localization of HSF1 to nuclear stress bodies at the single-cell level was negatively correlated with both HSF1 transcriptional activity and cell survival after stress in human osteosarcoma cells [31]. 

A key factor in the emergence of tumors is the evasion of senescence, permitting tumor cell immortalization and continuous proliferation [78]. HSF1 appears to be intimately connected to the senescence pathways. For instance, during senescence, HSF1 becomes inactivated through a pathway connected with a loss of SIRT1 deacetylase activity [79]. SIRT1 deacetylates HSF1 at K80, a key residue in HSF1 required for DNA binding and transactivation [80,81]. HSF1 actions also intersect with the p53 pathway to determine cell fates, including survival, programmed cell death, or senescence. For example, depletion of HSF1 has been shown to lead to cell senescence and to prevent malignant transformation [64,82]. These studies showed HSF1 depletion led to an increase in p21, which Oda et al. demonstrated to be mediated by increased *DHS2* expression and p53 stabilization in diploid human fibroblasts and to be independent of altered HSP levels [82]. HSF1 may also modulate senescence by activating Hsp72 expression, levels that were shown to be key factors in overcoming oncogene-induced senescence [83,84]. HSF1 also co-operates with p53 in interactions involving HSF1 co-complex with p53 and can augment transcriptional activation of p53 gene targets in response to genotoxic stress [85]. The complex relationship between HSF1 and p53 was also reflected by a study that showed HSF1 to influence clonogenic growth based upon whether the cells expressed wild-type p53 or a p53 mutant variant [66]. 

### 2.2. HSF1 Modulates Cell Cycle Progression and Mitosis

Improper cell cycle progression and cell proliferation are fundamental features of tumorigenesis and common in cancer cells whose innate cell cycle regulatory mechanisms are characteristically lost to some degree [78]. These changes ultimately lead to a greater capacity for rapid rates of cell division and poor fidelity in genome replication and a subsequent increased probability for further genomic aberrations. Multiple studies have shown HSF1 to affect cell cycle progression, where manipulation of HSF1 activity or expression alters the distribution of cell populations across different stages of the cell cycle [64,85,86,87,88,89]. In an apparent pro-homeostatic regard, HSF1 promotes senescence or G2 arrest after thermal or genotoxic stress and facilitates DNA repair [85,86,87]. HSF1 was also shown to localize to the spindle poles in metaphase and to be phosphorylated at S216 by Plk1 during early mitosis, an event stabilized by Cdc20. Upon release from Cdc20, HSF1 is ubiquitinated for degradation by SCP^β-TrCP^, an event that was necessary for mitotic progression [88]. Of note, while this study demonstrated that degradation of HSF1 was necessary for mitotic progression, HSF1 deletion or knockdown caused defective mitosis, thereby indicating temporal functions for HSF1 throughout the cell cycle. Bruce et al. reported a temporal increase in HSF1 binding to DNA upon the entry of quiescent cells into G1 phase, and overexpression of HSF1 promoted the proportion of G1 cells, an effect also observed upon overexpression of an HSF1^L22^ mutant [89]. HSF1 may also regulate the cell cycle through a series of downstream gene targets associated with cell cycle progression, including *CDC6, CKS2,* and *CKS1B* [39]. Considered together, it appears that the loss of HSF1 is a cell condition that promotes senescence and/or limits cell cycle progression in the absence of cellular stress. Under acute stress, HSF1 acts to limit cell cycle progression and promotes senescence, a likely pro-homeostatic response that may protect against transformation. However, in transformed contexts, such as in the presence of active oncogenes HER2, Ras, or mutant p53, increased HSF1 activity enables cancer cells to overcome senescence and cell cycle checkpoints and thereby enhances the capacity for tumorigenesis.

Most normal and malignant cells respond to mitogenic growth factors (GFs), and HSF1 appears to be required for downstream signaling from a range of GFs, including the heregulin receptor Her3 (ERBB3), the ligandless Her2 receptor, and the platelet-derived growth factor receptor (PDGFR) [45,60,63,64]. These are key GFs involved in the growth of multiple normal and malignant cells, suggesting that HSF1 may be required to permit the growth signals transmitted through GF receptors. Consistent with this property, reduced proliferation rates were reported in *Hsf1*^+/*−*^ and *Hsf1^−/−^* murine mammary tissue compared to *Hsf1^+/+^* tissues and were linked to *Hsf1* deletion reducing activation of the mitogenic ERK1/2 pathway downstream of TGF-β treatment and either Neu or Ras expression [63]. In addition, HSF1 was also shown to support constitutive activation of the MAPK pathway due to loss of *NF1* [90].

### 2.3. Aneuploidy

HSF1 has been shown to induce aneuploidy by altering the chromosomal distribution during mitosis [91]. This form of genomic instability provides cancer cells the opportunity to reshuffle chromosomes and chromosomal fragments, leading to increased growth rates or drug resistance. Aneuploidy is often associated with decreased cell differentiation and increased tumor malignancy. HSF1 influences this process by binding CDC20 and blocking CDC20–CDC27 interaction. This interaction decreases activation of the anaphase-promoting complex (APC) and results in incomplete mitosis [92]. CDC20–HSF1 interaction is governed by PLK1 phosphorylation of HSF1 at S216 [88], and effective p53 activity is required for this HSF1-driven phenomenon [93]. HSF1 interaction with Shugoshin may also influence the proper chromosomal distribution [94]. Overall, these findings hint at how the overexpression of HSF1 could confer a proliferative advantage. Severe aneuploidy, however, may lead to the depletion of HSF1 and could compromise HSP90 chaperone activity [95], suggesting that certain cancer cells may be susceptible to therapies that further disrupt the protein-folding capacity. 

### 2.4. Cell Growth and Energy Metabolism

In contrast to most normal tissues, tumor cells show a characteristic shift in energy metabolism, with glycolysis coming to dominate over oxidative phosphorylation, a transformation known as the Warburg effect, named after the Nobel prize-winning cell biologist Otto Warburg [96]. The Warburg effect seems to give the tumor cells a growth advantage in the rapid generation of ATP through glycolysis. A number of studies have indicated that the elevated HSF1 levels in cancer cells can modulate energy metabolism and switch glucose utilization towards the glycolytic pathway [45,97,98]. This ability to alter metabolism may reflect an evolutionarily conserved process, based on the need for expedient ATP production via glycolysis after stress and the need to avoid engaging possibly damaged mitochondria and their pro-apoptotic components, a protective process that could also benefit the emergence of cancer. In non-transformed contexts, HSF1 appears to have a powerful effect upon cellular energy stores, including ATP and NAD+ levels [98]. HSF1 was found to modulate NAD+ levels through positive regulation of the *NAMPT* gene, which encodes a key factor of the NAD+ salvage pathway [98]. The effect of HSF1 depletion upon ATP and NAD+ levels was more pronounced in a fasting state and led to reduced Sirtuin-1 activity and widespread protein acetylation [98]. In both cancer and non-cancer cells, HSF1 activity is also subject to the energy status of the cell. Fasting leads to repression of HSF1-mediated HSP expression in liver tissue and hepatoma cells through an apparently direct regulatory action of Peroxisome proliferator-activated receptor γ coactivator 1α (PGC-1α), a central coordinator of metabolic programs [99]. In contrast, under heat stress, PGC-1α augments HSP expression and thermal tolerance [100]. HSF1 also has a reciprocal regulatory action upon PGC-1α, as it binds to the *PGC-1α* promoter and can positively regulate its gene expression [101]. A mechanistic basis for the regulatory interplay between PGC-1α and HSF1 upon gene expression has been demonstrated by several studies that have shown PGC-1α and HSF1 to co-complex in cells, directly interact in binding assays, and to each recognize the HSE DNA-binding motif [99,100,102]. HSF1 was also shown to be acutely responsive to translational flux and is positively regulated by the mTOR kinase, a key coordinator of protein translation [7,103]. Conversely, loss of HSF1 was found to lead to increased AMPK phosphorylation and decreased mTOR activity [98,104]. The flux of nuclear HSF1 was also found to match the nutrient availability [98]. While the studies of Santagata et al. showed that inhibition of protein translation inhibited HSF1 activity and did not affect total HSF1 expression levels, Bruce et al. found that HSF1 levels increased upon entry to G1 phase, and also reported the translation inhibitor cycloheximide to inhibit HSF1 binding to DNA [89,103]. Therefore, HSF1 is both affected by rates of metabolism and is an upstream regulator of metabolic flux. 

### 2.5. HSF1 and Tumor-Initiating Cells (TICs)

Most tumors contain a population of cells capable of initiating tumors and metastases [105]. Such TICs resemble tissue stem cells in terms of their ability to populate new generations of daughter cells and in their patterns of gene expression and cell surface markers [54,106]. TICs sorted from the tumor population in MMT murine breast cancer were found to be enriched in activated HSF1 phosphorylated at S326 as well as in downstream products, such as Hsp72 and MTA1 [107]. HSF1-phospho S326 was enriched approximately a 1000-fold in the mouse mammary TIC sorted by the expression of surface markers Sca-1^+^ and CD44^+^, compared to non-TICs, which possessed a Sca1^−^CD44^−^ phenotype. Knockout of the elevated Hsp72 in these cells markedly reduced metastasis [107]. In addition, HSF1 was involved in the stem cell phenotype in human breast carcinoma cells, such as MDA-MB-231 and MCF7, which were rich in HSF1 phosphorylated on the activating site, S326, and low in HSF1 phosphorylated on the repressive site, S303 [59]. HSF1 phosphorylation on S326 involved the kinase mTOR and led to an increase in nuclear β-catenin, a powerful inducer of stem cell proliferation. Knockdown of either HSF1 or mTORC1 abolished the tumor-initiating potential of human mammary carcinoma cells as indicated by a reduced capacity to form tumor spheroids in vitro [59], consistent with the study by Carpenter et al., who also reported reduced tumor spheroid formation under conditions of HSF1 inhibition [108]. Hsf1 was also shown to be an important factor for the emergence of an EMT phenotype in experimental tumor cells driven by the *Neu* oncogene or TGF-β treatment [63], as well as through control of the EMT regulator Slug downstream of HRG and HER2 [109]. The biochemical processes that regulate and define the TIC phenotype remain areas of intense research, although activation of the EMT program is generally recognized to be involved in promotion of the TIC potential of carcinoma cells. Compared to more differentiated counterparts, TICs are characterized to be situated within the stem-like or progenitor populations of a tumor that also possess distinct epigenetic organization and metabolic preferences [110,111,112]. HSF1 may also have a potential regulatory role upon TIC potential through its activities in metabolic coordination and/or association with DNA-modifying enzymes, such as histone deacetylases HDAC1 and HDAC2, DNMT3a, or the SWI/SNF complex [113,114,115]. 

### 2.6. HSF1 Contributes to Changes in the Local Tissue Environment to Support Tumorigenesis

In addition to deregulated cellular processes that confer enhanced tumorigenic properties to carcinomas cells, significant changes occur within the local tissue environment that facilitate tumorigenesis. Tumor growth depends on the ability to establish de novo microcirculation, as oxygen and nutrients have a limited ability to diffuse though unperfused layers of tissues. HSF1 was shown to play a role in angiogenesis through its effect on the oxygen-sensing transcription factor HIF1-α, which stimulates angiogenesis in response to hypoxia [46]. This is likely related to the role of HSF1 in increasing the expression of HSP90, which chaperones HIF1-α and maintains its intracellular levels [116] along with supporting the activity of receptor tyrosine kinases, such as VEGFR, that signal for neovascularization [117]. HSF1 also appears to act indirectly on HIF-1α through the RNA-binding protein HuR, as discussed below [46]. Consistent with these studies, Xi et al. also reported reduced vascularization in *Hsf1^+/−^* and *Hsf1^−/−^ Neu*-driven mammary tumors compared to *Hsf1^+/+^ Neu*+ tumors, based upon CD31 staining [63]. With roles in both angiogenesis and the Warburg effect, HSF1 therefore appears to exert a major influence on the bioenergetics of tumor cells. 

### 2.7. Metastasis

To achieve metastatic growth, cancer cells must successfully invade surrounding tissues, intravasate into the microcirculation, become lodged in the vascular beds of distant tissues, extravasate, and then sustain growth and invasion of the colonized tissues. It is a highly a complex and multifactorial process and is the primary cause of cancer morbidity [118]. HSF1 appears to be closely involved in the metastatic cascade as indicated by multiple studies [37,38,63,119,120,121]. The potent influence of HSF1 on metastasis may involve downstream effects on the tumor cells mediated through HSPs [107,122,123] or may involve novel transcriptional targets for HSF1 [38,39]. One such HSP-independent mechanism for HSF1 involves its binding to metastasis-associated factor 1 (MTA1). MTA1 can broadly affect gene expression as a co-repressor or co-activator through its chromatin-modifying activities, including as a component of the nuclear remodeling and deacetylation (NuRD) multiprotein complex, and can influence several processes associated with tumorigenesis, as reviewed in [124]. For example, HSF1-MTA1 complexes were involved in repressing estrogen receptor (ER)-regulated genes, a stage in the switch from more benign estrogen-dependent luminal breast cancers to the more metastatic Her2 phenotype [125,126]. The HSF1–MTA1 interaction may be a mechanism by which HSF1 epigenetically reprograms the genome to drive a cancer-specific transcriptional program [39]. The NuRD complex may also in some circumstances have *trans*-activating properties that contribute to HSF1 activation. Another mechanism involves the binding of HSF1 phosphorylated at serines 303 and 307 to the ubiquitin ligase FBXW7: Loss of this factor in cancer is associated with an amplification of HSF1 levels and increased metastasis [127,128].

Another key factor in promoting tumor growth and metastasis, in addition to tumor cell intrinsic alterations, is the influence of the extracellular tumor stroma that is recruited to support and maintain the cancer cells in situ [55,129]. The most notable factors in the pro-tumor stroma are the cancer-associated fibroblasts (CAFs) that disrupt normal tissue structure and secrete collagen fibers and growth-promoting factors [130,131]. CAFs in human breast cancer biopsies were shown to be rich in HSF1, and the depletion of HSF1 led to a loss of the factors transforming growth factor-β (TGF-β) and CXCL12/SDF1 known to program the tumor microenvironment in a pro-tumorigenic and metastatic manner [50]. These factors act in concert to maintain a pro-metastatic immune-suppressed tumor microenvironment that favors tumorigenesis [132]. Other tumor-infiltrating cells, such as tumor-associated macrophages (TAMs), may also be dependent on HSF1 activity to maintain high levels of HSPs, whereby Hsp72 was shown to be important for TAM infiltration of tumors [133]. TAMs are known to secrete growth factors favoring tumor growth. 

The observation that CAFs exhibit increased levels of HSF1 despite not harboring overexpressed or mutated oncogenes poses significant questions concerning oncogenesis, including how does HSF1 become overexpressed in normal and malignant tissues? A circuitous mechanism can be proposed here, in which HSF1 upregulates CXCL12/SDF1 levels by directly binding its gene promoter, while increasing TGF-β levels through indirect mechanisms [50]. Secretion of CXCL12/SDF1 and TGF-β by CAFs promotes tumorigenesis [129], and TGF-β increases HSF1 levels in tumor cells [134]. This process involves upregulating the expression of FAM3C and YY1 [135], through a mechanism likely involving the stabilization of mRNA transcripts [136]. FAM3C is known to promote the epithelial–mesenchymal transition (EMT) [137], and YY1 has been found to bind within the POLR2A footprint of the *HSF1* promoter [138]. Together, these observations describe a positive feedforward loop, in which HSF1 and TGF-β induce the expression of the other factor in tumor cells and CAFs. This amplification of HSF1 expression and activity could trigger a chronic state of stress and enable tumorigenesis. How cancer cells instigate the expression of TGF-β and HSF1 in CAFs is another key question. Work with cardiomyocyte stem cells has shown that heat-shocked/stressed Sca-1^+^ cells produce exosomes containing HSF1 protein that are received by other cardiomyocytes to provide protection from ischemia [139]. This observation hints at the possibility of stressed TICs shedding HSF1-loaded exosomes to be taken up by CAFs, which in turn program the expression and secretion of TGF-β and CXCL12/SDF1 to further promote tumorigenesis. Consequently, monitoring exosomes for their content, including non-coding RNAs, may provide insight into how tumor cells manipulate the surrounding stroma and TAMs [140,141,142].

### 2.8. Resistance and Recurrence

In addition to metastasis, tumor resistance to treatment and tumor recurrence are major hurdles to achieving the desired clinical outcomes. Given the difficulty of modeling recurrence with experimental tumor models, the importance of HSF1 to tumor recurrence can currently be at best inferred from its association with the CSC phenotype in the tumor cell population. In this regard, various indicators for increased HSF1 activity have been found to associate with reduced relapse-free survival and are often increased in cells exhibiting markers for a CSC phenotype [47,52,59,143,144]. Of note, however, a recent study demonstrated inducible knockout of *Hsf1* to protect mice against NOTCH1-driven leukemia without recurrence for more than one year [47].

Several studies have shown that HSF1 also modulates the sensitivity of cultured cancer cells to a variety of cytotoxic agents [65,145,146]. This effect may be at least in part connected to the pro-survival properties that HSF1 confers to cells as discussed above. Several non-*HSP* gene targets of HSF1 that promote resistance and/or reduced sensitivity to cytotoxic agents have been identified, including *MDR1* and *DEDD2* [65,147,148]. HSF1 and activation of the HSR has also been associated with enhanced radioresistance [87,149,150], a relationship potentially linked to co-operation between HSF1 and the DNA repair machinery [87,151], and/or altered cell cycle dynamics [150]. Compounds shown to inhibit HSF1 activity, such as NZ28, were found to increase the radiosensitivity of cultured cancer cell lines, an effect that was augmented upon co-treatment with the HSP90 inhibitor NVP-AUY922 or 17AAG [150,152]. NVP-AUY922 was also shown to preferentially sensitize lung cancer cell lines to radiation in combination with HSF1 knockdown [146]. Cells exhibiting an increased CSC potential characteristically have an enhanced DNA repair capacity, and as mentioned above, increased indicators for active HSF1. CD44+/Sca1+ cells that were shown to have enhanced radioresistance and increased HSF1 activity were also found to be induced after radiation treatment [107,153,154]. HSF1 may coordinate resistant cancer cell properties through chromatin organization in cooperation with HDACs or other DNA-modifying complexes, such as MTA1, each of which have also been linked to resistance to cytotoxic compounds [155,156]. Alternatively, HSF1 has also been shown to promote resistance to cytotoxic therapies through its pro-glycolysis metabolic regulatory activities [51]. A recent study also demonstrated Hsf1 to be a capacitator for the phenotypic diversity across yeast cell populations, and that heterogenous Hsf1 regulation of Hsp90 was essential for the emergence of anti-fungal resistance [157]. Findings from this study introduced the concept that a greater range for dynamic control of Hsf1 activity via phosphorylation rather than extended or constant high levels of Hsf1 activity as the primary factor enabling Hsf1-mediated resistance. 

## 3. Regulation of HSF1 Activity in Cancer and Post-Transcriptional Pausing

With continued identification of the multifaceted ways in which HSF1 facilitates tumorigenesis, it has become an attractive target for cancer therapy. Key to the development of an HSF1 inhibitor suitable for clinical use is gaining a comprehensive understanding of the mechanistic basis for HSF1 activation in cancer. The simplest and most immediate hypothesis for HSF1 activation in cancer is that cells undergo proteotoxic stress during tumorigenesis, in a milder recapitulation of the heat shock response. As oncogenes become mutated and overexpressed, they may unfold and would require continuous chaperoning by Hsp90 and periodic refolding by other chaperones [158,159]. Thus, cancer cells would experience an increased folding demand. This demand would be predicted to influence *HSP* gene transcription initiation and elongation. Other factors, such as metabolic and translational flux, as discussed above, also appear to have a dominant effect upon HSF1 activity. Significant advances have been made in identifying HSF1 target genes, its upstream regulators, and the sequence of events leading to its regulation of target genes. However, the primary causes for altered HSF1 activity and expression in cancer remain to be fully established. In addition, the mechanisms by which HSF1 regulates its target genes, including interactions with transcriptional machinery and chromatin-modifying proteins, also remain to be fully defined. 

### 3.1. HSF1 Gene Structure and Regulation in Cancer

**Figure 4:**
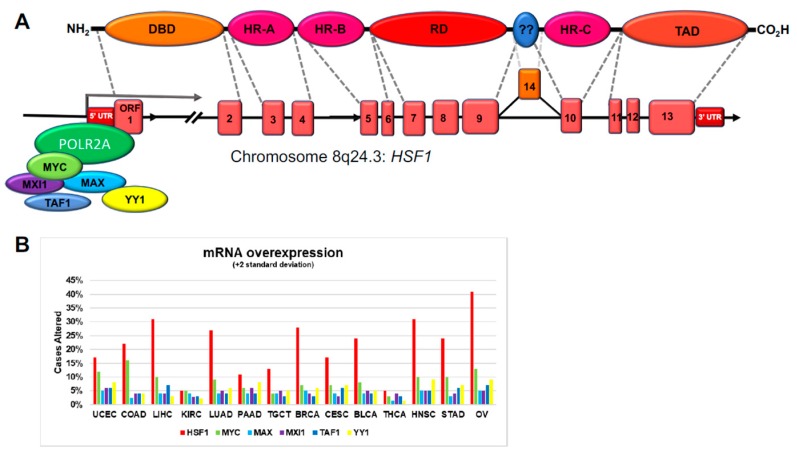
HSF1 functional domains and encoding gene structure, and amplification frequency of genes encoding *HSF1* transcriptional regulators across human cancers. (**A**) Structure of the human *HSF1* gene based on mRNA transcript (NM_005526.4) and protein (NP_005517.1). DBD; DNA-binding domain, HR-A; hydrophobic repeat A, RD; regulatory domain, TAD; transcriptional activation domain. Several transcription factors are found to bind within the RNA polymerase II (*POLR2A*) transcription start site (TSS) footprint according to ENCODE [138]. Data attained from the UCSC Genome Browser [161]. (**B**) HSF1 is overexpressed in a higher percentage of cancer cases across tumor types compared to other transcription factors, such as MYC. Data attained from cBioportal (OQL script “Gene: EXP>2”) [162,163].

HSF1 mRNA levels are frequently elevated across a larger percentage of cancer cases, as suggested by tumor biopsy analysis, although as mentioned above, the biological processes that regulate *HSF1* gene expression are yet to be fully determined (Figure 2A). According to ENCODE ChIP-seq data aggregated by the UCSC Genome Browser [138], five transcription factors were consistently observed within the RNA Polymerase II (POLR2A) footprint encompassing the transcription start site (TSS). These included MYC and its cohorts MAX and MXI1 along with TAF1 and YY1 (Figure 4A). *MYC* is a well-studied oncogene and is encoded in the same genomic neighborhood as *HSF1.* Ironically, data from The Cancer Genome Atlas show that *HSF1* mRNA levels are elevated across most tumor types as compared to MYC and its partners (Figure 4B). This may reflect the high significance of HSF1 in promoting oncogenesis. A direct oncogenic link between HSF1 and MYC likely exists as HSF1 is required for MYC-driven hepatocarcinogenesis [160]. YY1 is also found within the *HSF1* promoter and may increase HSF1 levels in response to TGF-β [135]. NOTCH1 was also shown to directly regulate *HSF1* mRNA in T-ALL [47]. 

*HSF1* is encoded at chromosome 8q24.3, a genomic hotspot for several cancers, including prostate, liver, and bladder [164,165,166]. The gene structure of human *HSF1* is composed of 13 exons, with one alternatively spliced-in exon between the RD (Exon 9) and HR-C/LZ4 (Exon 10). Germline analysis indicates that Exon 9 possesses the most variation, which encodes the C-terminal end of the RD [167]. This includes the missense SNP (rs78202224, P365T) that is associated with increased breast cancer incidence [168]. However, compared to other oncogenic factors, the structure of HSF1 appears to be maintained with high fidelity in a range of cancers, perhaps suggesting the need for intact HSF1 in transformation and tumor progression (Figure 2G–I).

### 3.2. Molecular Chaperone Feedback Interaction

One of the major hypotheses regarding HSF1 activation is that the factor is constitutively repressed by its products Hsp70 and Hsp90, an elegant pathway of feedback inhibition [169,170], although a recent report questions the inferences of these early studies [171]. What is now becoming apparent is that each HSP regulates HSF1 in a unique manner. Hsp70 and the constitutively expressed paralog Hsc70 bind all along the unstructured domains of HSF1 and can interact with both the “closed” monomer and “open” trimer conformations of HSF1 [171]. These data suggest that Hsp70 and Hsc70 may repress HSF1 in multiple ways, including: (i) The binding and stabilization of inactive monomers, (ii) disassembly of active trimers, (iii) blockade of key transcription-related protein–protein interactions by binding to the TAD. 

Hsp90, on the other hand, seems to only bind when HR-A/B and HR-C are dissociated and HSF1 is in an “open” conformation. The Hsp90–HSF1 interaction is indeed transient and requires Hsp90 to be locked in its own “closed” conformation to be consistently observed by immunoprecipitation Western blot analysis, thus demonstrating that Hsp90 ATPase activity greatly influences HSF1 interaction. This “closed” Hsp90–HSF1 interaction, however, is disrupted by ATP-competing small molecules that target the N-terminal domain of HSP90, such as Ganetespib (STA-9090) [171]. These findings suggest that Hsp90 regulates HSF1 by disassembling active trimers and that N-terminal Hsp90 inhibitors increase HSP production in part by preventing attenuation of the HSR. This may go some way to explaining why Hsp90 inhibitors have not been effective in treating cancer, due to their role in inducing the protective stress response [172]. The stress-inducible paralog, Hsp90α, interacts with HSF1 more strongly than constitutively expressed Hsp90β. Hsp90α also prefers to bind near the HR-A/B trimerization domains while Hsp90β tends to bind the RD and HR-C domains [171]. Hsp90β binding may also alter HSF1 thermosensitivity [173]. In addition, phosphorylation of HSF1 at S121 in the HR-A domain increases Hsp90 interaction and reduces transcriptional activity [174]. Considered together, these findings indicate that Hsp90α and Hsp90β regulate HSF1 activity by controlling the rate and lifetime of leucine-zipper trimerization. 

HSF1 activity is also negatively regulated by the TRiC/CCT complex [175]. In malignant cancer cell lines, HSF1 is reported to bind the gene promoters of multiple TRiC subunits [39]. TRiC complexes are typically known to chaperone cytoskeleton components [176] but have recently been found to associate with Cajal bodies and regulate telomerase activity, thereby influencing cell mortality [177]. 

Throughout cancer progression, oncogenes are mutated and/or overexpressed. HSF1 feedback inhibition is therefore thought to be disrupted by client oncogenes sequestering HSPs and other regulating chaperones, thus leading to increased HSF1 activity. However, some evidence suggests that sufficient protein-folding capacity is available within cancer cells. Instead, alterations in cell signaling pathways dependent on HSP activity may enable and promote oncogenesis [123,178]. HSF1 and HSPs are heavily post-translationally modified (reviewed in [36,179,180,181]). In cancer, abnormal modification of HSPs could alter interactions with and regulation of HSF1, while an aberrant HSF1 modification states may drive malignant growth. Analysis of the *PhosphoSitePlus* proteomic database hints at this possibility, through its profiling of thousands of cancer-related samples [182]. Post-translational modifications are another layer of code that dictate cell biology. Further deciphering the post-translation modification code that governs HSF1 activity may provide critical clues. 

### 3.3. Post-Transcriptional Pausing

The *Hsp70* gene was the first to be shown to be regulated at the level of post-transcriptional pausing, a process in which RNA polymerase was shown to become paused a few base pairs down from the TSS still attached to an RNA transcript [183,184]. HSF1 binding to the promoters of *HSP* genes leads to the relief of pausing and permits transcriptional elongation in a mechanism involving recruitment of the elongation factor pTEFb and the mediator complex [183]. Phosphorylation of HSF1 on S320 by PKA positively influences this process [185,186]. There is, however, very little information regarding the role of this mechanism, or its subversion in cancer. Another potentially relevant study showed a global role for Hsp90 in the relief of pausing and transcriptional elongation [187]. One could envisage a role for HSF1 in amplifying the rate of elongation in cancer through transcription of *HSP90* and triggering of this mechanism. Another interesting possibility is the potential role of DNA double strand breaks (DSBs) and topoisomerase II in HSF1-mediated transcription [188]. HSP gene transcription involves the generation of DSBs through topoisomerase II cleavage and artificially induced DSBs were able to trigger or allow transcription [26,188]. DSBs were shown to track with POLR2A in the open reading frames of genes during transcription, suggesting an intriguing link between DSB, the DSB signaling response, and *trans*-elongation in the context of the cancer cell in which DNA repair is often compromised [26]. It also provides questions concerning transcription-induced mutagenesis. One intriguing question that still remains and is highly pertinent is the exact molecular mechanisms’ coupling of HSF1 binding to HSE-containing promoters and the relief of stalled elongation complexes.

### 3.4. Co-Operation with Other Factors to Activate Transcription and Influence the Transcriptome

HSF1 has been shown to co-operate with several other co-factors to modulate its biochemical activities. For example, HSF1 can form heterotrimers with HSF2 that may enable the tailoring of HSF1 activities to contextual cues as well as potentially diversify its DNA recognition sites [189,190]. Matrix metalloproteases play key roles in invasion and metastasis by breaking down extracellular matrix molecules and permitting tumor cell invasion and motility [191]. Recent studies indicate that MMP3 can also function within the cell and trigger HSP expression in collaboration with HSF1 and chromatin-binding CBX3 [192]. It may be informative to examine this non-canonical role for MMP3/HSF1 in mammary cancer in which MMP3 is expressed to high levels. 

HSF1 can also mediate its effects upon the cancer transcriptome indirectly through the actions of the protein products of its target genes, for example, the RNA-binding protein HuR [46]. This protein binds to the 3′UTR sequences of a wide range of mRNAs, many of whose translated protein products play key regulatory roles in tumorigenesis. The binding of HuR to mRNA generally leads to its stabilization and thus to elevated mRNA levels, but it can also cause enhanced translation. HuR induction appears to be on the HSF1>HSP signaling track and involves Hsp70 [46,59]. HSF1-mediated HuR increases are involved in β catenin accumulation in human mammary TIC by enhancing translation as well as in pro-angiogenic HIF1 induction. As HuR binds to a broad array of target mRNAs, its participation downstream of HSF1 is likely to lead to increases in mRNAs other than those encoding the classical HSPs [59]. 

### 3.5. Pathways Downstream of HSF1

By analogy with the heat shock response, one would envisage key roles in tumorigenesis for its products, the HSPs, as downstream effectors of HSF1 [193]. There is indeed considerable evidence for the participation of many of the HSPs in cancer, particularly Hsp27, Hsp70, and Hsp90, and the TRiC/CCT chaperonins, which are elevated in many tumor types and play roles in most of the processes that determine the tumor phenotype, including growth, evasion of death and senescence pathways, invasion, metastasis, and stemness [11,57,193]. However, chromatin immunoprecipitation combined with next generation sequence analysis (ChIP-Seq) carried out on a range of tumor cells and tissues have shown recently that HSF1 also associates on chromatin with a cohort of genes that are not of the classical *HSP* family [39]. This conclusion is not entirely at odds with findings in the study of the heat shock response, which show an HSF1 association with both *HSP* and non-*HSP* as well as the binding of factors other than HSF1 to chromatin after stress [184]. However, comparing the transcriptomes of tumor cells with those of heat-shocked cells indicated marked differences in the transcriptional properties of the constitutively activated HSF1 found in advanced cancer [39]. Genes avidly induced by heat shock, such as HspA6, were only moderately induced in transformed cells. Ontological analysis of the novel group of genes associated with HSF1 and transcribed in cancer cells with a metastatic phenotype included Ly6K and prominin 2, which are associated with stem cell properties and metastasis. Overall, the group of genes marked by abundant HSF1 association included groups associated with protein folding and the stress response, mRNA translation, cell cycle progression and proliferation, and DNA repair [39]. Activation of these genes generally involved association with HSE within the promoter regions. These data suggested that HSF1 activation in cancer is qualitatively different to its activation in the stress response, that the factor is redirected to novel targets, although the exact molecular differences in regulation are not clear. HSF1–HSE binding seemed to be involved, suggesting that events secondary to DNA binding, such as association with novel transcriptional regulatory proteins, may also play a role. HSF1 can associate with the NuRD complex as well as complexes containing the histone acetylase p300 and it would be informative to determine potential roles for these factors in HSF1 functions in cancer at the level of histone modification [38,186,194]. Further analysis of the HSF1-regulated signature genes uncovered by Mendillo et al. [39] in a range of human tumors suggested a remarkable clustering of the most frequently expressed genes on chromosome 8q [195]. The significance of this finding, however, awaits elucidation.

## 4. Targeting of HSF1 with Drugs

Given its robust roles in enabling oncogenesis and its apparent dispensability to non-transformed tissues, HSF1 appears an attractive therapeutic target. Numerous small molecule drugs have therefore been developed that aim to reduce HSF1 activity, although most agents have not been shown to directly interact with HSF1 and have yet to show sufficient preclinical potential to enter the cancer clinic [48,196]. The development of specific HSF1 inhibitors has been challenged by the limited opportunities for compound target sites within HSF1 crystal structures solved to date. Although, recent structural model studies identified a hydrophobic groove between the HSF1 DBD helices α1 and α2 that may serve to facilitate an interaction with the wing loop of alternate HSF1 DBDs bound to HSEs containing more than two inverted 5-bp units [197]. Given that co-operation between subunits of an HSF1 trimer facilitates the trimer–DNA interaction [198], targeting this region of HSF1 may perturb the affinity of HSF1 trimers to HSE motifs [197]. Based on the preferential affinity of HSF trimers for specific HSE arrangements [198], any compound that does successfully target the HSF1 DBD may have differential effects upon HSF1 binding to distinct HSEs. The flexibility exhibited by the linker region between the DBD and HR-A/B, as well as that of the RD and TAD, and the difficulties in crystalizing these latter regions are major challenges for the development of specific HSF1 compound inhibitors. Nevertheless, a small molecule compound screening approach was recently shown to yield the HSF1 inhibitory compound I_HSF1_115, which directly interacts with the HSF1 DBD and is cytotoxic to cultured cancer cells [199]. 

For the present time at least, the targeting of upstream activators, modulators, and downstream effectors of HSF1 may be the most effective way to contain its oncogenic activity. For example, one approach may be targeted activation of HSF1 negative regulators, such as p300/CBP, which acetylates HSF1 at K80 and thereby reduces the positive charge of this residue and its affinity for the negatively charged DNA phosphate backbone [197,200]. Conversely inhibiting SIRT1, a molecule that deacetylases HSF1 at K80 [200], may also comprise a similar strategy for targeting HSF1 DNA binding. Kourtis et al. found oncogenic NOTCH1 in T-ALL to significantly increase *HSF1* mRNA expression and to co-operate with HSF1 to transactivate a cohort of *HSP* genes. Increased *HSF1* mRNA in primary T-ALL patient samples and cultured cells could be effectively targeted by γ-secretase inhibition and subsequent blockade of NOTCH1 activation [47]. The study demonstrated cancer-type-specific inhibition of HSF1 by targeting an upstream oncogene activator, an approach that may apply to other tumor contexts where oncogenic drivers lead to HSF1-mediated increased chaperome expression [47]. Of note, the study also showed that knockdown of *HSF1* or several individual *HSP* genes significantly reduced the survival of cultured human T-ALL cells, and that HSF1-dependent survival could not be rescued by overexpression of *HSP90AB1* alone [47]. These findings suggest the importance of at least some of the individual *HSP* genes to survival in this model to be related to the functionality of the chaperome network. In addition, the finding emphasizes the potential effectiveness of targeting the chaperome by inhibiting HSF1. Another approach to target HSF1 may be to favor its degradation by approaches, such as facilitating FBXW7-mediated HSF1 ubiquitylation and proteolysis [127]. Developing agents that specifically lock HSF1 on to its regulating chaperone complexes may also be a worthwhile strategy. However, further progress in this area will require a more complete understanding of HSF1 biology. 

RNA interference may be another method that could be employed to specifically target HSF1. The frequent amplification of *HSF1* mRNA expression across many cancer types (Figure 2) [47,65] would provide higher levels of the target for any delivered homologous RNAi molecule compared to normal tissue, and thereby possibly increase the chances of a greater fold reduction of HSF1 expression. As discussed above, HSF1 activity is not primarily regulated at the mRNA level, although given evidence that HSF1 can drive malignancy [50], and that in some contexts, mRNA levels are associated with patient outcome (Figure 2F), a substantial reduction of *HSF1* mRNA that leads to reduced protein activity may have potential for significant therapeutic benefits. Although, the use of RNA interference of oncogenic factors in cancer therapy is currently undergoing a great degree of scrutiny [201]. Recent reviews discuss the current status of HSF1 inhibitory molecules in greater depth [48,196].

## 5. Perspective

Our understanding of the mechanisms of HSF1 activation have advanced considerably since HSF1 was first identified as the regulator of HSP genes. Crystal structures of HSF1 complexed with DNA motifs have revealed specific interactions between HSF1 and DNA and HSF1 with other HSFs [189,197,202]. However, several features of HSF1 activation still remain to be established. These include how the HSF1 transcriptional activation domain uncouples transcriptional pausing, the mechanism by which the regulatory domain inhibits HSF1 activity, as well as the primary factors leading to HSF1 activation in cancer cells. Given the relative lack of tertiary order exhibited by the regulatory and TAD domains, resolving further details of HSF1 activation related to these domains may continue to be inhibited by the difficulty of obtaining crystal structures for these regions. 

Our analyses further demonstrate *HSF1* mRNA levels to be frequently increased in human tumor tissue relative to normal tissues. The root cause for this also remains undetermined but may in a subset of tumors be fueled by amplified MYC activity, or by NOTCH1 in the case of T-ALL [47]. Of note, *HSF1* mRNA levels also positively correlated with many *HSP* mRNAs whether HSF1 promoter occupancy was observed or not. This observation suggests the possibility of common higher-order mechanisms of increased *HSF1* and *HSP* gene expression in such cancers, including permissive chromatin rearrangements, copy number variation (e.g., ovarian cancer, Figure 2 and Figure 4), or common regulators, such as the discussed NOTCH1 [47]. We reviewed several non-transformed and transformed contexts where HSF1 knockout can lead to dramatic changes in bioenergetics and cellular fate. This might suggest some adverse consequences in normal tissue for its targeting in cancer. However, the general wellbeing of *Hsf1^−/−^* mice, and the observed apparent normal hematopoiesis achieved by *Hsf1*-depleted murine hematopoietic stem and progenitor cells would suggest that an HSF1-targeted therapy could be reasonably well tolerated [47,68]. As the primary factor in molecular chaperone expression, HSF1 activation is a proteostatic strategy for several aging diseases, and further insights into the mechanism of HSF1 activation would provide important advances for endeavors to therapeutically modulate its activity in both cancer, neurodegeneration, and aging [203].

**Figure 5:**
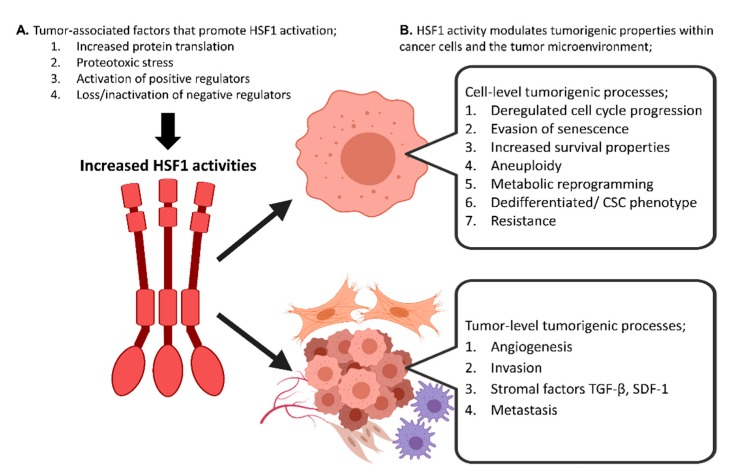
Summary of factors leading to HSF1 activation in cancer, and subsequent tumorigenic processes fostered by HSF1 activity. (**A**) Several features characteristic of cancer cells can lead to increased HSF1 activity. These include, as listed on the left of the figure, increased protein translation; proteotoxic stress; activation of HSF1 activators, such as MYC, Ras, mTOR, and Sirt1; and loss of negative regulators, including NF1, and FBXW7. (**B**) HSF1 has been shown to enable several cell-intrinsic tumorigenic processes, including the deregulation of cell cycle progression, aversion of senescence, cell survival, aneuploidy, metabolic reprogramming, dedifferentiated/CSC phenotypes, and resistance. In addition, HSF1 activity can modulate several tumorigenic features that manifest at the tumor tissue/microenvironmental level, including angiogenesis, invasion, or stromal activation. HSF1 has also been linked to the formation of metastatic tumors, possibly as a product of the above activities and/or through additional mechanisms yet to be elucidated. Figure created with Biorender.com.

Overall, the general pathological contributions of HSF1 during tumorigenesis appear to involve a combination of facilitating tumor growth by the fostering of growth signals and evasion of death pathways, as well as the modulation of metabolic programming, features of a pro-tumorigenic tumor microenvironment, and the TIC phenotype (Figure 5). Therefore, controlling HSF1 and the stress response may ultimately be an important factor in successfully treating some cancers. Although HSF1 activates the classical *HSP* genes during stress, it also leads to the repression of a large number of genes [184]. In addition, many genes are either activated or repressed in a non-HSF1-dependent manner in response to heat shock and the significance of this finding for tumorigenesis is yet to be fully understood. In addition, in cancer, many of the genes bound on chromatin by HSF1 are non-*HSP* genes [39]. The respective roles of HSF1 in the stress response and cancer are thus complex and not fully explainable by the currently available data, although a thorough grasp of this area is essential for consideration of this factor as a target in cancer. Additionally, efforts should be made to investigate several other aspects of HSF1 biology, including the significance of the HSP–HSF1 interaction dynamics and post-translational modifications on the overall transcriptional activity. Moreover, this analysis should be applied to HSF paralogs, HSF2 and HSF4, which directly interact with HSF1. Next, whole-exome sequencing efforts suggest the need to explore the role of genetic variation within the HSF1-signaling axis in predisposing individuals to cancer. Finally, it would be desirable to elucidate the mechanisms that allow for the amplification of HSF1 expression and activity in both tumor cells and CAFs.
